# Seasonal Migration in the Aphid Genus *Stomaphis* (Hemiptera: Aphididae): Discovery of Host Alternation Between Woody Plants in Subfamily Lachninae

**DOI:** 10.1093/jisesa/ieaa103

**Published:** 2020-09-30

**Authors:** Tetsuya Yamamoto, Mitsuru Hattori, Takao Itino

**Affiliations:** 1 Interdisciplinary Graduate School of Science and Technology, Shinshu University, Asahi, Matsumoto, Nagano, Japan; 2 Graduate School of Fisheries and Environmental Sciences, Nagasaki University, Bunkyo, Nagasaki, Nagasaki, Japan; 3 Department of Biology and Institute of Mountain Science, Shinshu University, Asahi, Matsumoto, Nagano, Japan

**Keywords:** Lachninae, heteroecy, morphological comparison, mtDNA phylogeny

## Abstract

About 10% of aphid species show host alternation. These aphids migrate between primary and secondary host plant species in spring and autumn. Host alternation has not been observed in subfamily Lachninae, although it has been suggested on the basis of circumstantial evidence that *Stomaphis japonica* (Takahashi) may alternate its host between *Quercus serrata* (Murray) and *Quercus acutissima* (Carruth). However, a molecular phylogenetic study has indicated that the *Stomaphis* individuals feeding on these two plant species belong to two different lineages and aphids feeding on *Q. acutissima* and *Pinus densiflora* (Sieb. & Zucc.) belong to the same lineage. Here, we examined host alternation in *Stomaphis* species by comparing molecular phylogenetic identities, morphological features, and life cycles. The molecular analysis and morphological examination showed that aphids feeding on *Q. acutissima* were the same as those feeding on *P. densiflora*, whereas aphids feeding on *Q. serrata* were different from those feeding on *Q. acutissima* or on *P. densiflora*. Furthermore, winged aphids were observed on both *Q. acutissima* and *P. densiflora* in autumn, but we did not observe winged aphids on *Q. serrata*. These results indicate that *Stomaphis* (Walker) individuals feeding on *Q. serrata* and *Q. acutissima* belong to two species, one that feeds year-round on *Q. serrata,* and another, heteroecious species that feeds on *P. densiflora* as a primary host and on *Q. acutissima* as a secondary host. This study documents host alternation in subfamily Lachninae for the first time and discusses the acquisition of host alternation by *Stomaphis* from evolutionary and ecological perspectives.

Some species of aphids (Hemiptera: Aphididae) seasonally change host plants during their life cycles. This host alternation may be adaptive against inducible plant defenses or it may allow better utilization of plant nutrients (Kennedy and Stroyan 1959, Dixon 1971). However, only about 10% of aphid species exhibit seasonal host alternation (heteroecy). These species use woody plants as their primary host (where they reproduce sexually) and herbaceous plants as a secondary host (where they reproduce asexually; Dixon 1998, Blackman and Eastop 2006). Typically, host alternation in aphids occurs as follows.

In autumn, wingless (apterous) sexual females (oviparae) mate with winged (alate) males on the primary host plant and produce overwintering eggs. In all other life cycle generations, the aphid morphs are parthenogenetic, viviparous females. In spring, each aphid that hatches from an egg (fundatrix) founds a clonal colony on new leaves. Descendants of the fundatrix remain on the primary host until the second or third generation, when adults develop wings. The winged adult ‘spring migrants’ disperse and colonize plants of the secondary host species. They remain on the secondary host during the summer, producing both alate and apterous females (virginoparae), until the onset of short days induces the production of ‘autumn migrants’, winged morphs that relocate to plants of the primary host species.

Two hypotheses to explain the evolution of host alternation in aphids have been proposed. According to the first hypothesis, host alternation is an adaptation to seasonal changes in the nutritional value of the phloem sap of host plants (Dixon 1998). The phloem sap of woody plants is more nutritious for aphids in spring than in summer. In spring, when woody plants leaf out, the phloem sap contains nitrogen, a high-quality nutrient; in summer, however, leaf growth stops as the plants invest in reproduction and the phloem sap contains an inadequate level of nitrogen. In contrast, the nutritional quality of the phloem sap of herbaceous plants in summer is higher, compared with that of woody plants, because herbaceous plants grow throughout summer. Therefore, aphids can achieve better population growth by migrating from woody plants to herbaceous plants in summer (Kundu and Dixon 1995). The second hypothesis posits that host alternation is a consequence of phylogenetic constraints. In autumn, aphids that alternate hosts must return to woody plants before overwintering because the fundatrix and sexual generations are constrained phylogenetically to woody plants (Moran 1988). The two hypotheses are not mutually exclusive; both assume that host alternation is an adaptation to take advantage of high-quality nutrients provided by herbaceous plants in summer.

Host alternation has been documented in four (out of 24) aphid subfamilies: Anoeciinae, Aphidinae, Eriosomatinae, and Hormaphidinae; moreover, the reconstructed aphid phylogeny shows that it has evolved twice (Ortiz-Rivas and Martínez-Torres 2010). No species in subfamily Lachninae (giant aphid group), the most basal clade of the Aphididae, have been considered to exhibit host alternation (Moran 1988, 1992, 1994; Jousselin et al. 2010; Ortiz-Rivas and Martínez-Torres 2010; Hardy et al. 2015). However, among Lachninae, *Stomaphis japonica* (Takahashi) (Takada 2008), *Pyrolachnus pyri* (Buckton) (Long and Chen 1988), and the *Nippolachnus piri* (Matsumura) species complex may have heteroecious life cycles (although the *N. piri* species complex may actually be not heteroecious, but oligophagous; Higuchi and Miyazaki 1969, Kanturski et al. 2018) Although these might be key species for understanding the evolution of host alternation in aphids, they have received little attention in such studies, perhaps because their life cycles have not been well documented.

*Stomaphis* (Walker) is the second largest genus in subfamily Lachninae; thirty-three species and three subspecies have been described worldwide (Blackman and Eastop 2020). *Stomaphis* aphids have large bodies and mouthparts about twice as long as their bodies. The very long proboscis and stylet allow for suction of phloem sap from tree trunks. Most *Stomaphis* species are described as mono- or oligophagous species that live on the same tree year-round (monoecy). Molecular phylogenetic analyses of 12 *Stomaphis* species (*S. abieticola* Sorin, *S. aceris* Takahashi, *S. aphananthae* Sorin, *S. fagi*, *S. hirukawai* Sorin, *S. japonica* Takahashi, *S. malloti* Sorin, *S. matsumotoi* Sorin, *S. pterocaryae* Sorin, *S. takahashii* Sorin, *S. ulmicola* Inoue, and *S. yanonis* Takahashi) distributed in Japan have revealed that most lineages and sublineages show specificity to particular host plant species (Yamamoto et al. 2020b). However, there are several exceptions among *Stomaphis* species found in Europe, including an anholocyclic species (*S. acquerinoi* Binazzi; Binazzi and Pennacchio 2005) and a species that uses a very wide range of host plants (e.g., *S. wojciechowskii* Depa; Depa et al. 2017). In addition, all *Stomaphis* species have a mutualistic relationship with partner ants, from which they receive protection from aphid predators and sanitary services in return for providing honeydew to the ants (Lorenz and Scheurer 1998). *Stomaphis* aphids exhibit these mutualistic relationships almost exclusively with ants of genus *Lasius* (Fabricius), suggesting that tending by *Lasius* ants is necessary for the survival of *Stomaphis* colonies (Yamamoto et al. 2020b). The strict dependence of *Stomaphis* on ant mutualism has been reported to cause evolutionary host plant shifts (Depa et al. 2017, Yamamoto et al. 2020b).

*Stomaphis japonica* is distributed in Japan, China, and Korea, and uses certain *Quercus* (L.) (Fagaceae, Fagales) species as host plants (Takahashi 1960, Seo 1994, Qiao and Zhang 1999, Takada 2008, Blackman and Eastop 2020, Yamamoto et al. 2020a, 2020b). The first record of *S. japonica* (as *S. quercus japonica*) is a description of apterous and alate viviparous females on *Q. acutissima* (Carruth) by Takahashi (1960). Takada (2008) described the *S. japonica* life cycle and reported possible host alternation between *Quercus serrata* (Murray) trees, as the primary host, and *Q. acutissima* trees, as the secondary host, but his observations were sporadic (noncontinuous). As a caveat, Takada (2008) mentioned that although alate viviparous females were observed to migrate from the secondary host (*Q. acutissima*) to the primary host, they were not observed on the primary host (*Q. serrata*) in autumn.

Yamamoto et al. (2020b), on the one hand, reported that *Stomaphis* aphids feeding on *Q. acutissima* and those feeding on *Q. serrata* belong to different molecular phylogenetic lineages, suggesting that the reported host alternation between *Q. serrata* and *Q. acutissima* is not probable. On the other hand, Takada (2008) reported that *Stomaphis* aphids on *Q. acutissima* could not be observed in spring and autumn, suggesting that host alternation may indeed exist in *Stomaphis* aphids. Yamamoto et al. (2020b) also reported that *Stomaphis* aphids feeding on *Q. acutissima* and those feeding on *Pinus densiflora* (Sieb. & Zucc.) (Pinaceae, Pinales) belong to the same molecular phylogenetic lineage. Therefore, to clarify life cycles of *Stomaphis* aphids in Japan, it is necessary to examine not only the life cycle on each host plant but also to compare the genetic and morphological characteristics of aphids feeding on *Q. acutissima*, *Q. serrata*, and *P. densiflora*.

In this study, we aimed to clarify life cycles of a heteroecious species in Lachninae, in which heteroecy is rare. To confirm host alternation in *S. japonica*, we compared morphology, mtDNA genetics, and life cycles of *S. japonica* aphids feeding on *Q. serrata*, *Q. acutissima*, and *P. densiflora* in Kyoto (the study area of Takada 2008) and other areas in Japan.

## Materials and Methods

### Field Sampling

**Fig. 1.**
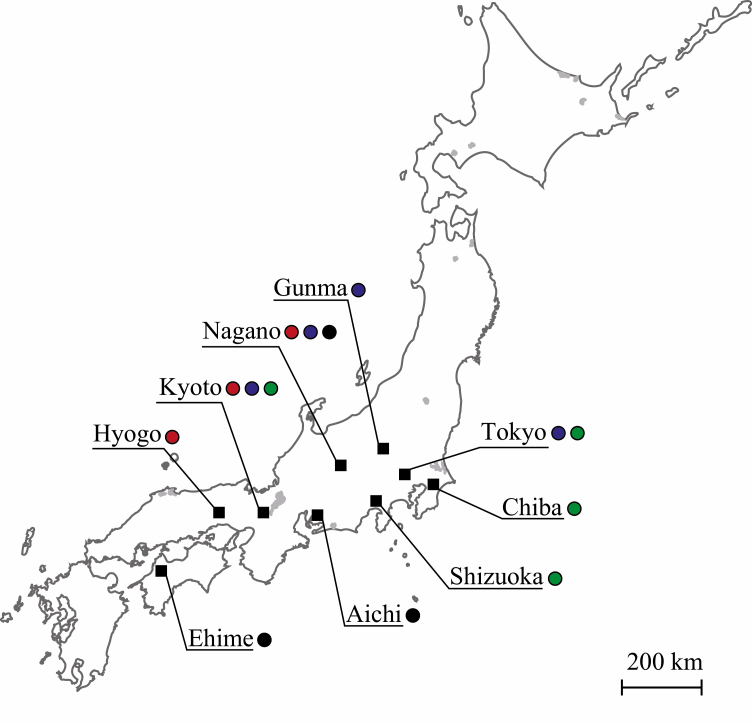
Sites in Japan where *Stomaphis* aphid colonies were observed and sampled. The black boxes indicate collection points. The plant species from which aphids were collected are shown by colored circles: red, *Pinus densiflora*; blue, *Quercus acutissima*; green, *Q. serrata*; black, other.

We found 50 *Stomaphis* aphid colonies on five host plant species, *Q. acutissima*, *Quercus variabilis* (Blume), *Q. serrata*, *P. densiflora*, and *Picea jezoensis* var. *hondoensis* (Mayr), at nine sites (Aichi, Chiba, Ehime, Gunma, Hyogo, Kyoto, Nagano, Shizuoka, and Tokyo) in central and western Japan by searching for known host plant species and by tracking *Lasius* ant trails (Supp Tables S1 and S2 [online only]; Fig. 1). We regarded all aphids on a single host plant as belonging to a single colony. Up to five aphid individuals including at least one adult were collected from the 50 colonies and stored at 4°C in 99.5% ethanol until DNA extraction for phylogenetic analysis. Aphid specimens for morphological comparison were collected from eight of the 50 DNA-extracted aphid colonies and from one colony without DNA extraction (so, 9 colonies in total) and stored in 70% ethanol until they were mounted on slides (K3-K56). All DNA samples and mounted slides have been deposited in the Department of Biology, Faculty of Science, Shinshu University.

### Aphid DNA Extraction and Sequencing

Total genomic DNA was extracted from a single aphid of each colony by using a DNeasy Blood & Tissue Kit (Qiagen, Hilden, Germany) following the manufacturer’s instructions. We targeted the molecular marker mitochondrial cytochrome oxidase c subunit II (*COII*). The *COII* gene was amplified by PCR with Takara Tks Gflex (Takara Bio, Shiga, Japan) using the PCR primer set mt2993+ (5′-CATTCATATTCAGAATTACC-3′) and Eva-R (5′-GAGACCATTACTTGCTTTCAGTCATCT-3′; Brower and Jeansonne 2004, Stern 1994). The PCR temperature profile was 30 cycles of 98°C for 10 s, 50°C for 10 s, and 72°C for 60 s. After amplification, the PCR product was purified with ExoSap-IT* (USB, Cleveland, OH) and sequenced by using a BigDye* Terminator version 1.1 Cycle Sequencing Kit (ABI, Weiterstadt, Germany) on an ABI 3130 Genetic Analyzer.

### Phylogenetic Analysis

The mitochondrial *COII* sequences were edited and aligned to 623 bp with SeqScape v. 2.5 software (ABI, Weiterstadt, Germany). Four *Stomaphis* species (*S. fagi*, *S. hirukawai*, *S. ulmicola*, and *S. yanonis*) collected by H. Yoshitomi were used as outgroups. We constructed a neighbor-joining phylogenetic tree based on the Tamura-Nei + gamma model with MEGA7 software (Kumar et al. 2016) to determine aphid lineages. The robustness of the tree was assessed by nonparametric bootstrapping with 1000 replicates.

### Morphological Analysis

For morphological analysis, we used all collected adult aphids of each colony. Aphid specimens were immersed in 10% KOH and encapsulated in Canada balsam by the method of Kozarzhevskaya (1986). We took pictures of each aphid slide and used ImageJ (Schneider et al. 2012) to measure morphological features. We measured the lengths of antennal segments I to VI, the processes terminalis (PT), the primary rhinarium (PR), and the first and second segments of the middle (MT) and hind tarsus (HT) of all morphs. We also calculated ratios between selected features (Antenna I/II, III/II, III/IV, V/IV, VI/V and PT/Antenna VI, PT/PR, HTI/MTI, HTII/MTII, HTI/HTII, and MTI/MTII). The morphological features and their measurements and the calculated ratios are shown in Supp Tables S3–S6 (online only). Morphological identification of each sample was performed by T.Y. with reference to a key of Japanese *Stomaphis* species (Sorin 2012). We performed a principal component analysis (PCA) with R software ver. 3.5.2 (R Development Core Team 2018) for each morph (apterous vivipara, alate vivipara, ovipara, and fundatrix) using the ratios of the morphological features. Then we compared the mean first principal component (PC1) score between aphid populations feeding on *Pinus densiflora* and *Q. acutissima* and populations feeding on *Q. serrata* to determine whether the populations were morphologically separable.

## Results

### Field Sampling

We collected 44 colonies from the three *Quercus* species, five colonies from the *P. densiflora*, and one colony from *Picea jezoensis* var. *hondoensis* (Mayr) Rehder (Supp Tables S1 and S2 [online only]; Fig. 1). In all seasons and areas, aphids sucked phloem sap from the bark surface on the lower trunks of the trees and all aphid colonies were attended by *Lasius* spp. or *Camponotus obscuripes* (Mayr) ants. With regard to their geographic distribution, *Stomaphis* aphid colonies dwelling on *Q. acutissima* were found in Gunma, Kyoto, Nagano, and Tokyo; colonies dwelling on *Q. variabilis* Blume were found in Aichi and Ehime; those dwelling on *Q. serrata* were found in Chiba, Kyoto, Shizuoka, and Tokyo; and those dwelling on *Pinus densiflora* were found in Hyogo, Kyoto, and Nagano (Fig. 1). In Kyoto, where Takada (2008) reported host alternation of *S. japonica* between *Q. serrata* and *Q. acutissima*, we collected *Stomaphis* individuals from three plant species (*Q. acutissima*, *Q. serrata*, and *P. densiflora*).

### Phylogenetic Analysis

**Fig. 2.**
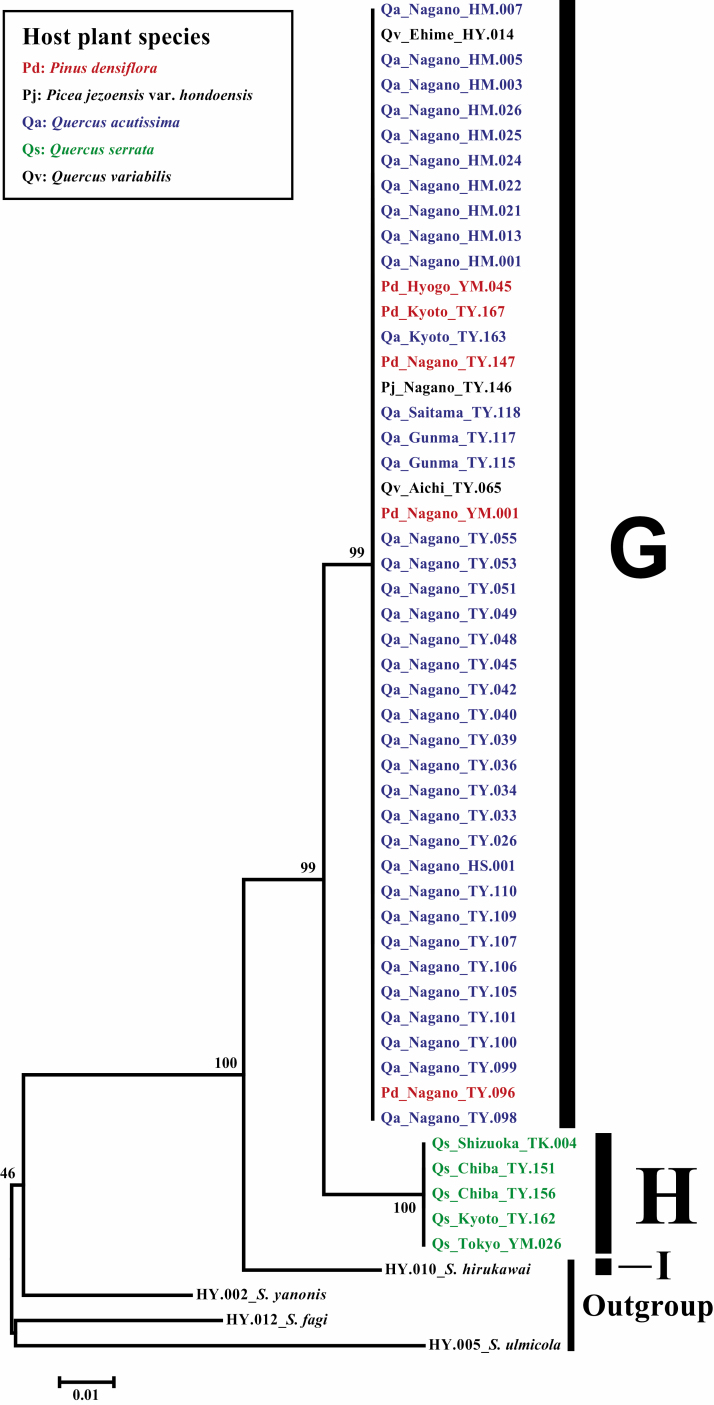
Neighbor-joining phylogenetic tree of *Stomaphis* aphids feeding on *Quercus acutissima*, *Q. serrata*, *Q. variabilis*, *Pinus densiflora*, and *Picea jezoensis* var. *hondoensis*, based on a partial DNA sequence of the mitochondrial *COII* gene. The phylogenetic tree shows two distinct lineages (Lineages G and H). For each operational taxonomic unit, the host plant species, collection site, and DNA voucher number are shown. See Supp Table S1 (online only) for details of the specimens. The bootstrap probability is shown for each node, and the scale indicates a nucleotide substitution rate of 0.01.

The partial *COII* gene obtained from the *Stomaphis* aphids consisted of 570 nucleotide sites, of which 54 were parsimony informative. The topology of the neighbor-joining tree revealed two well-resolved ingroup lineages (bootstrap values > 99%) designated G and H, following Yamamoto et al. (2020b) (Fig. 2). The genetic distance of *COII* between lineage G and H was 2.8%. Lineage G comprised individuals feeding on *Q. acutissima*, *Q. variabilis*, *P. densiflora*, and *Picea jezoensis* var. *hondoensis*, whereas lineage H included only individuals feeding on *Q. serrata*. No genetic differentiation within lineage G was detected, either geographically or with respect to host specificity.

### Morphological Analysis

**Fig. 3.**
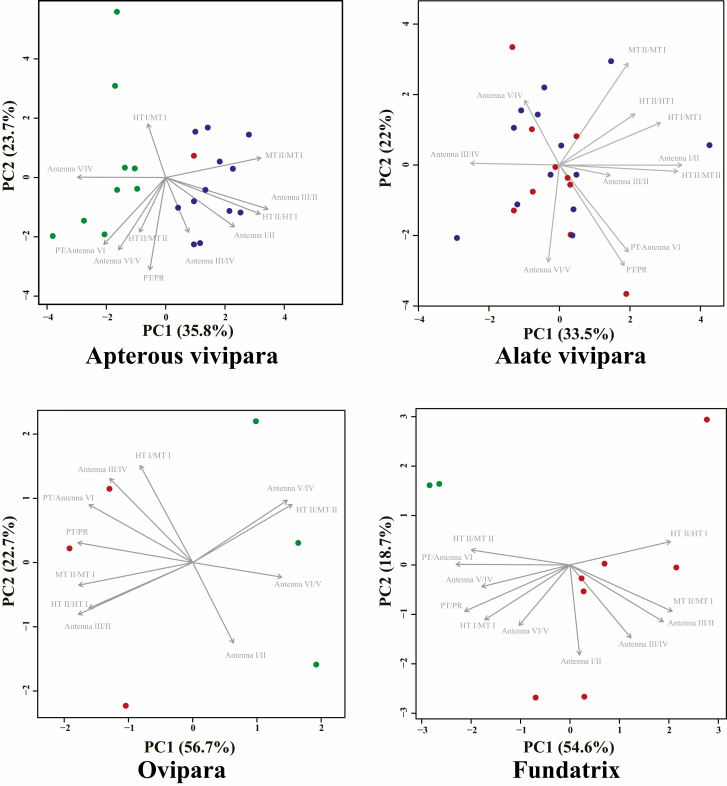
Principal component analysis results based on ratios of morphological features in each aphid morph. Plot colors indicate the plant species from which the specimen was collected: red, *Pinus densiflora*; blue, *Quercus acutissima*; green, *Q. serrata*. The contribution ratio of each principal component is indicated on each axis. The arrows show the factor loadings on the first and second principal components (PC1 and PC2) of each ratio of morphological features. See Supp Table S2 (online only) for a list of the specimens used in the morphological analysis, and Supp Tables S3–S6 (online only) for the detailed measurement results.

In all aphid morphs, the PCA results for the 11 morphological traits showed that plot clusters of aphid individuals feeding on *Q. acutissima* and *Pinus densiflora* overlapped with each other but not with plot clusters of aphid individuals feeding on *Q. serrata* (Fig. 3). The mean PC1 scores differed between aphids dwelling on *Q. acutissima* or *P. densiflora,* and aphids dwelling on *Q. serrata* in apterous viviparae (*t* = 9.51; df = 21; *P* < 0.01), oviparae (*t* = 7.68; df = 21; *P* < 0.01), and fundatrices (*t* = 3.97; df = 21; *P* < 0.01). The mean PC1 scores did not differ between aphids dwelling on *Q. acutissima* and aphids dwelling on *P. densiflora* in alate viviparae (*t* = 0.43; df = 21; *P* = 0.67).

The principal component loadings showed that the length ratios antenna I/II, antenna III/II, MT II/MT I, and HT II/HT I were good discriminating parameters in apterous viviparae. The antenna III/II ratio was clearly different between the individuals feeding on *P. densiflora* and *Q. acutissima* (minimum 5.52, maximum 7.14, mean 6.23), and individuals feeding on *Q. serrata* (minimum 3.53, maximum 5.09, mean 4.38; Supp Table 3 [online only]). Values of the other ratios overlapped among individuals feeding on different host plants.

All parameter ratios in alate viviparae overlapped between individuals feeding on *P. densiflora* and individuals feeding on *Q. acutissima* (Supp Table 4 [online only]). There were some nonoverlapping parameters in oviparae and fundatrices between individuals feeding on *P. densiflora* and individuals feeding on *Q. serrata*, but these comparisons were based on only a few specimens (Supp Tables S5 and S6 [online only]).

### Life Cycles on Each Host Plant

#### *Stomaphis* Aphids on *P. densiflora*

**Fig. 4.**
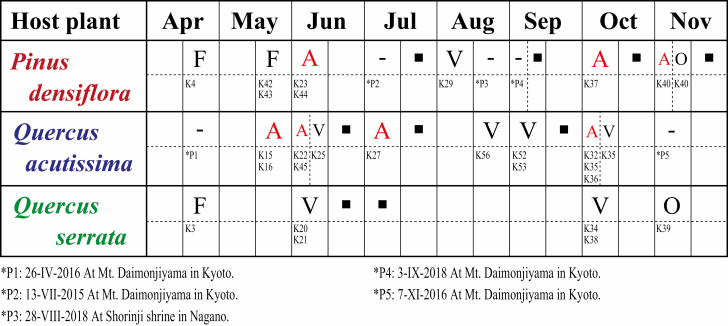
Observed occurrence pattern of *Stomaphis* aphids on three host species: *Pinus densiflora*, *Quercus acutissima*, and *Q. serrata*. Morphs of aphids collected in the first and second half of each month are shown above the dashed line in each row. Morphological voucher numbers are shown below the dashed line (see Supp Table S2 [online only] for details). Morph abbreviations: F, fundatrix; V, apterous vivipara; A, alate vivipara; O, oviparous females and males. Alate viviparae (A) that migrate between host plants are shown in red. Black boxes indicate periods when DNA samples were collected and analyzed but the morph was unknown. Hyphens indicate that observations were conducted but no aphids were observed; dates and locations are given in footnotes (*P1–*P5).

*Stomaphis* individuals were found on *P. densiflora* trees from early April to early June and from early October to early November. They were not found in late July or early September at Mt. Daimonji (Kyoto) or in late August at Matsumoto (Nagano) (Fig. 4). Overwintered eggs hatched in early April, and fundatrices were found in late April. Adult fundatrices and nymphal fundatrigeniae (virginoparae produced by fundatrices) were found in late May. The fundatrigeniae became alate adults in early June. Subsequently, all of these alate viviparae dispersed to somewhere. In Kyoto and Nagano, aphid individuals were not observed from July to September, except at Kisohukushima (K29) in Nagano, where aphid individuals were observed in August. The aphid colony in Kisohukushima consisted of fewer than 10 individuals, however. Alate viviparae appeared in Kyoto and Nagano in October, having migrated from somewhere. These alate viviparae were slender immediately after flying to *P. densiflora* from somewhere, but their abdomens became enlarged after they started to suck phloem sap from the trunk. Oviparae and males were probably produced by the alate viviparae (sexuparae) from late October to November. Because we did not conduct continuous observations, however, we did not observe alate viviparae giving birth to oviparae and males, but we confirmed that alate viviparae and the sexual morphs were living together in the same colony. In November, mating was observed.

#### *Stomaphis* Aphids on *Q. acutissima*

*Stomaphis* individuals were found on *Q. acutissima* from late May to early October in Tokyo, Gunma, Nagano, and Kyoto, but they could not be found in other months (Fig. 4). From late May to early July, alate viviparae arrived on *Q. acutissima* trees, having migrated from somewhere, and subsequently produced apterous viviparae. From July to September, the adult apterous viviparae produced mainly apterous nymphs, but some alate viviparae produced by the apterous viviparae (observed in July) were found in summer. Aphid colonies consisting of more than 100 individuals were frequently observed during the summer. In early October, the alate viviparae observed on *Q. acutissima*. The second to fourth instars of alate viviparous nymphs sucked phloem sap from *Q. acutissima*, but when they became alate adults, they stopped exhibiting the sucking behavior and migrated as slender alate adults to somewhere. No aphid individuals were observed in November. Fundatrices, oviparae, and males were not observed on *Q. acutissima* at any time.

#### *Stomaphis* Aphids on *Q. serrata*

*Stomaphis* individuals that occurred on *Q. serrata* passed through a holocycle (Fig. 4). In early April, overwintering eggs and then fundatrices were observed. Apterous viviparae, but no alate viviparae, were observed from early June to early October. From late October to early November, oviparae and males were observed, which mated and laid eggs on the trees to overwinter.

## Discussion

**Fig. 5.**
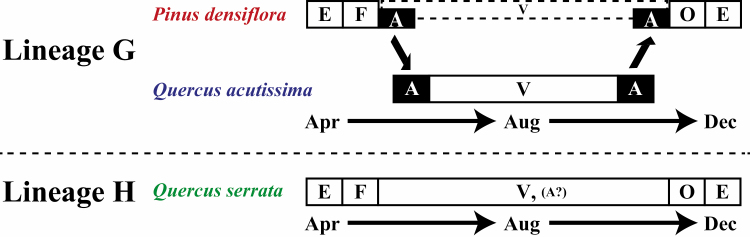
Schematic life cycle of the two *Stomaphis* lineages. Morph abbreviations: E, overwintering eggs; F, fundatrices; V, apterous viviparae; A, alate viviparae; O, oviparous females and males.

We compared phylogeny, morphology, and life cycles among *Stomaphis* individuals using different hosts. Although Takada (2008) recognized aphids feeding on *Q. serrata* and *Q. acutissima* as one species, our comparisons revealed that these *Stomaphis* individuals could be divided into two lineages. On the basis of our observations of aphid life cycle on the different host plant species, we found that one lineage alternated between *P. densiflora* as primary host and *Q. acutissima* as secondary host, whereas the other lineage used only *Q. serrata* throughout year (Figs. 4 and 5). The results of the morphological comparison showed that in apterous viviparae, the ratio of the third to the second antennal segment was higher in the lineage that alternated hosts than in the lineage without host alternation, but no morphological differences were found in alate viviparae within the lineage with host alternation (Fig. 3). Furthermore, the phylogenetic analysis based on the mitochondrial *COII* gene revealed no phylogenetic variation among collecting sites within either lineage, but 2.8% of the base pairs of the lineage with host alternation differed from those of the lineage without host alternation (Fig. 2). This genetic distance between the two lineages suggests that they are distinct at the species level, because the average intraspecific divergence of the mitochondrial *COII* gene among *Stomaphis* species is about 0.4% (Chen et al. 2012).

Our results thus clearly confirm the existence of a heteroecious species in subfamily Lachninae, in which host alternation has not previously been recognized. Moreover, we obtained these results in Kyoto, where Takada (2008) suggested that *S. japonica* might alternate between *Q. serrata* trees as its primary host and *Q. acutissima* trees as its secondary host; therefore, the heteroecious life cycle of the *Stomaphis* lineage found in this study (*P. densiflora* as primary host and *Q. acutissima* as secondary host) is more likely to be correct than the life cycle inferred by Takada (2008). The observed seasonal occurrences of *S. japonica*-related *aphids on each host plant (Q. acutissima*, *P. densiflora*, and *Q. serrata*; Figs. 4 and 5) in this study are consistent with previous observations of *S. japonica* (or *S. pini*; see the discussion on systematics below; Takahashi 1960, Blackman and Eastop 1994, Takada 2008). With regard to the few aphid individuals observed on *P. densiflora* in summer at Kisohukushima (K29, Nagano; Figs. 4 and 5), it is possible that aphids at Kisohukushima use only *P. densiflora* throughout the year, because *Q. acutissima* is sparsely distributed there. Thus, the local population of *Stomaphis* aphids may not be able to use *Q. acutissima* as a secondary host.

### Evolution of Host Alternation in *Stomaphis*

Lineage G, which uses *Q. acutissima* and *P. densiflora* as alternate hosts, is the only reported example of host alternation in genus *Stomaphis*. In the overall molecular phylogeny of *Stomaphis*, lineage G occupies a relatively derived position (Yamamoto et al. 2020b), suggesting that acquisition of host alternation represents an exception. The existence of seasonal host alternation in *Stomaphis* is surprising because *Stomaphis* aphids on a single host tree are not considered to be nutritionally limited during any season (Depa 2013, Depa et al. 2015).

Aphids have diversified by shifting to novel host plant species (Peccoud et al. 2010). In general, similarities of morphological and physiological characteristics of phylogenetically closely related plant species allow evolutionary host shifts (Peccoud et al. 2010). On the other hand, host shifts between distantly related host plant species should be difficult for aphids because the aphids must develop novel adaptations to acquire new hosts. Interestingly, however, we found that lineage G uses two very distantly related hosts: *Q. acutissima* (Fagaceae, Fagales), an angiosperm, and *P. densiflora* (Pinaceae, Pinales), a gymnosperm. Lineage G is a sister group to lineage H, which feeds on *Abies firma* (Sieb. & Zucc.) (Pinaceae), *Q. serrata* (Fagaceae), *Quercus dentata* (Thunb.) (Fagaceae), and *Betula platyphylla* (Sukaczev) (Fagaceae), and lineages G and H together form a sister group to lineage I, which feeds on *Chamaecyparis obtusa* (Sieb. & Zucc.) (Cupressaceae, Pinales; see Yamamoto et al. 2020b). *Stomaphis cupressi* (Pintera) of subgenus *Parastomaphis* (Pašek), which occupies a more basal phylogenetic position than lineages G, H, and I, also uses gymnosperm trees (*Cupressus* spp., Cupressaceae, Pinales; Blackman and Eastop 2020). These relationships suggest that lineage G may have been preadapted to feed on Pinaceae (or Pinales) and Fagaceae species.

Of the two main hypotheses that have been proposed to explain the evolution of host alternation in aphids (Moran 1988, 1992, 1994, Jousselin et al. 2010, Hardy et al. 2015), the historical (phylogenetic) constraint hypothesis (Moran 1988), that is, ‘specialization of the fundatrix to the primary host’, is plausible, in some aphid groups at least. In aphids, it would presumably be adaptive for all generations to feed on the secondary host (without host alternation), because the nutrient value of the secondary host is generally higher than that of the primary host. However, according to this hypothesis, the specialization of the fundatrix to the primary host means that the next generation cannot be produced unless the aphids return to the primary host from the secondary host. This hypothesis is supported by evidence from three aphid subfamilies (Anoeciinae, Eriosomatinae, and Hormaphidinae). In these subfamilies, the fundatrices of heteroecious species use only a narrow range of plant taxa as the primary host (Moran 1988, Hardy et al. 2015). Aphids of these subfamilies form a gall on the primary host, and they must be specialized to this host because in order to be able to form the gall, they must have developed adaptations to overcome the immune response of the host (Hardy and Cook 2010). In this study, however, the heteroecious aphids of *Stomaphis* lineage G do not need to change their host utilization between primary and secondary host plants. In addition, phylogenetic relationships in *Stomaphis* show that inter-order/family host plant shifts including the fundatrix generation have occurred frequently (Yamamoto et al. 2020b). Thus, fundatrices of aphids in genus *Stomaphis* are not restricted to a specific taxonomic plant group, and host alternation in *Stomaphis* cannot be explained by the historical constraint hypothesis.

According to the second hypothesis, that host alternation is an ecological optimization of host plant use (Dixon 1971), host alternation represents the adaptation of all aphid generations to seasonal changes in the nutritional value of the host species (Kundu and Dixon 1995, Jousselin et al. 2010). Evidence supporting this hypothesis is found in subfamily Aphidinae. Aphidinae species have evolved host alternation repeatedly and independently. Furthermore, heteroecious species in Aphidinae are usually polyphagous (Hardy et al. 2015). It is unknown whether aphids of *Stomaphis* lineage G benefit nutritionally from seasonal host alternation in each season. However, because lineage G appears to be the only heteroecious *Stomaphis* lineage (Yamamoto et al. 2020b), host alternation in *Stomaphis* must have evolved only once, which suggests that frequent adaptive phylogenetic host shifting is implausible as an explanation for host alternation in this case. Furthermore, the lineage G aphids use both *P. densiflora* and *Picea jezoensis* var. *hondoensis* as primary hosts and both *Q. acutissima* and *Q. variabilis* as secondary hosts; thus, they are oligophagous, not polyphagous. Therefore, the ecological optimization hypothesis, which has been suggested to explain heteroecy in Aphidinae, cannot explain the evolution of host alternation in genus *Stomaphis*.

Host alternation can be dangerous for aphids (Dixon and Kundu 1994). For example, in the heteroecious aphid *Rhopalosiphum padi* (L.), the estimated success rate of autumn migration is only 0.6% (Ward et al. 1998). The success rate of host alternation of aphids may be lower in *Stomaphis* than in other aphid genera because *Stomaphis* aphids are remarkably larger in body size and have a longer proboscis than other aphids, characteristics that interfere with their ability to fly and, therefore, to migrate between hosts (Depa et al. 2015). Furthermore, *Stomaphis* aphids have an obligate mutualistic relationship with ant species of genus *Lasius* (Yamamoto et al. 2020b). Thus, even if *Stomaphis* aphids successfully migrate between hosts, the newly established aphid colony will not succeed unless it encounters a suitable ant species on the new host. As a result, it is difficult for most *Stomaphis* species to acquire host alternation. To acquire and maintain host alternation in *Stomaphis*, the migration success rate, or the benefits conferred by the secondary host, must be sufficiently large, to offset the reduction of fitness resulting from migration failures.

To acquire host alternation in *Stomaphis*, the following conditions are required: 1) plants of the secondary host species must grow near plants of the primary host species and 2) *Stomaphis* aphids must achieve sufficiently higher growth and reproductive rates on the secondary hosts. In Japan, *Pinus densiflora* and *Q. acutissima* are dominant tree species in secondary forests used for fuelwood and charcoal by village people, and they grow sympatrically across wide areas. Therefore, in many areas, it would be easy for aphids to explore and colonize these two plant species. Deciduous *Quercus* trees seem to be suitable as host plants of *Stomaphis* aphids of lineages G and H, because most deciduous *Quercus* species distributed in Japan can be hosts to lineages G and H (*Stomaphis* aphids of lineage G use *Q. acutissima* and *Q. variabilis,* and lineage H aphids use *Q. crispula*, *Q. dentata*, and *Q. serrata*; Yamamoto et al. 2020b). Aphid colonies found in summer on *Q. acutissima* trees in areas except for Kisohukushima (where *Q. acutissima* does not occur) were observed to have more than 100 aphids, whereas aphid colonies on *P. densiflora* (K29) observed in Kisohukushima in the summers of 2016 and 2018 were very small, consisting of only 10 adults and nymphs. This contrast suggests that the heteroecious lineage G aphids, by migrating to the secondary host, experience a gain in fitness that is sufficiently high to offset the costs of migrating. A future study comparing the performance of the aphids on each host plant in summer may provide insights into the ecological factors affecting the acquisition of host alternation by aphids.

In European *Stomaphis*, host shifts and the development of anholocyclic forms, populations, or lineages may have occurred during the last glacial period (Depa 2013). The fossil pollen record shows that *P. densiflora* (a primary host plant species of *Stomaphis* lineage G) has been distributed in Japan since the early Pleistocene (Yamada et al. 2014). In contrast, *Q. acutissima* (a secondary host plant species of *Stomaphis* lineage G) seems to have not been distributed in Japan during the last glacial period because the genetic diversity of *Q. acutissima* in Japan is very low; rather, it was probably introduced from the Asian continent to Japan after the last glacial period by human activities (Zhang et al. 2015, Saito et al. 2017). In this study, because we did not detect genetic differentiation in the *COII* region of the mtDNA of *Stomaphis* between regions where lineage G aphids alternated hosts and the region where they did not (Kisohukushima), it is likely that host alternation evolved in *Stomaphis* lineage G recently (<0.01 Ma). We hypothesize that lineage G aphids in Kisohukushima, where *Q. acutissima* is rare, did not acquire (or they lost) host alternation because of the absence of suitable secondary host plants. To test this hypothesis, future studies should clarify the genetic relationships among populations that alternate and ones that do not alternate their hosts by conducting a more detailed population genetic analysis. It should also be ascertained whether *S. japonica* aphids that feed on *Quercus* spp. in China and Korea also belong to lineage G.

### Systematics of *Stomaphis japonica* and *S. pini*

Takahashi (1960) described *S. japonica* as an aphid species feeding on *Q. acutissima,* and *S. pini* as a species feeding on *P. densiflora* in Japan. Seo (1994) and Qiao and Zhang (1999) recorded *S. japonica* on *Quercus* sp. in Korea and China. As a result, it has been thought that *S. japonica* uses only several *Quercus* species as host plants and that *Stomaphis* aphids using *P. densiflora* (*S. pini*) are different from *S. japonica* (Blackman and Eastop 2020). This interpretation may have arisen because biologists are apt to identify the species of *Stomaphis* specimens on the basis of the host plant on which the collected specimens were found. This approach to identifying species, however, led to a misunderstanding of *S. japonica* and its complex life cycle, in particular, its use of *Q. serrata* and *P. densiflora* as primary hosts and *Q. acutissima* is a secondary host. Similar problems of species identification and ecological understanding have also arisen with regard to certain European *Stomaphis* species (the identity of *Stomaphis betulae* (Mamontova) and *Stomaphis quercus* (L.), Depa et al. 2012; and the polyphagism of *S. wojciechowskii*, Depa et al. 2017).

Our results indicate that the *Stomaphis* aphids feeding on *P. densiflora* and those feeding on *Q. acutissima* belong to the same species, whereas the *Stomaphis* aphids feeding on *Q. serrata* are different from those feeding on *Q. acutissima*. Our PCA results for the length ratio of antennal segments 3 to 6 can clearly be directly compared with results obtained by Takahashi (1960) (Supp Fig. 1 [online only]); this comparison indicates that the aphids identified by Takahashi (1960) as *S. japonica* and *S. pini* cluster with the aphids observed to feed on *P. densiflora* and *Q. acutissima* in this study. Thus, *S. japonica* and *S. pini* are apparently synonyms, whereas *Stomaphis* aphids feeding on *Q. serrata* appear to belong to a new species. In Japan, the subspecies *S. pini takaoensis* is recorded as feeding on *Abies firma* (Sorin 2012), and *Stomaphis* aphids feeding on *Q. dentata* (lineage H-II in Yamamoto et al. 2020b) are genetically closely related to those feeding on *Q. serrata* (lineage H-I in Yamamoto et al. 2020b). Therefore, the classification of Japanese *Stomaphis* aphids remains uncertain. In the future, the classification of not only *Stomaphis* species but also species of other aphid taxa should be reviewed by taking account of their molecular phylogeny, ecological information, and morphological features.

## Conclusion

To clarify the actual life cycles of a heteroecious *Stomaphis* species in Lachninae, in which heteroecy is rare, we compared morphology and mtDNA sequences among populations feeding on three different host plant species and observed the life stages of *S. japonica* on each host species. Although a previous study suggested that *S. japonica* alternates hosts between *Q. serrata* and *Q. acutissima*, we found the aphids on these hosts to belong to two different lineages: a heteroecious group migrating seasonally between *P. densiflora* as primary host and *Q. acutissima* as secondary host, and a monoecious group feeding only on *Q. serrata* year-round. This host alternation in *Stomaphis* cannot be fully explained by the evolutionary patterns of host alternation in other Aphididae taxa. Therefore, more detailed population genetic analyses of heteroecious *Stomaphis* species would provide new insights into the evolution of host alternation in aphids.

## Supplementary Material

ieaa103_suppl_Supplementary_Table_S1

ieaa103_suppl_Supplementary_Table_S2

ieaa103_suppl_Supplementary_Table_S3

ieaa103_suppl_Supplementary_Table_S4

ieaa103_suppl_Supplementary_Table_S5

ieaa103_suppl_Supplementary_Table_S6

ieaa103_suppl_Supplementary_Figure_S1
