# Supplementary material for: Seasonal Migration in the Aphid Genus Stomaphis (Hemiptera: Aphididae): Discovery of Host Alternation Between Woody Plants in Subfamily Lachninae
Source: J Insect Sci. 2020 Sep 30;20(5):13. doi: 10.1093/jisesa/ieaa103 (PMC7583267; doi:10.1093/jisesa/ieaa103)
Supplement: ieaa103_suppl_Supplementary_Table_S1 [file ieaa103_suppl_supplementary_table_s1.docx]

Table S1. Aphid specimens used in the molecular phylogenetic analysis.

| no. | DNA voucher # | Lineage | Host plant | Collection date | Location | Latitude | Longitude | Collectors | Genbank #COII |
| --- | --- | --- | --- | --- | --- | --- | --- | --- | --- |
| 1 | HM. 001 | G | *Quercus acutissima* | 15-VIII-2014 | Kamiminochi, Nagano | - | - | H. Miyairi | LC519249 |
| 2 | HM. 003 | G | *Quercus acutissima* | 15-VIII-2014 | Kamiminochi, Nagano | - | - | H. Miyairi | LC519259 |
| 3 | HM. 005 | G | *Quercus acutissima* | 15-VIII-2014 | Kamiminochi, Nagano | - | - | H. Miyairi | LC519260 |
| 4 | HM. 007 | G | *Quercus acutissima* | 15-VIII-2014 | Kamiminochi, Nagano | - | - | H. Miyairi | LC519261 |
| 5 | HM. 013 | G | *Quercus acutissima* | 18-VIII-2014 | Matsumoto, Nagano | - | - | H. Miyairi | LC519251 |
| 6 | HM. 021 | G | *Quercus acutissima* | 22-VIII-2014 | Matsumoto, Nagano | - | - | H. Miyairi | LC519254 |
| 7 | HM. 022 | G | *Quercus acutissima* | 22-VIII-2014 | Matsumoto, Nagano | - | - | H. Miyairi | LC519255 |
| 8 | HM. 024 | G | *Quercus acutissima* | 22-VIII-2014 | Matsumoto, Nagano | - | - | H. Miyairi | LC519257 |
| 9 | HM. 025 | G | *Quercus acutissima* | 22-VIII-2014 | Matsumoto, Nagano | - | - | H. Miyairi | - |
| 10 | HM. 026 | G | *Quercus acutissima* | 22-VIII-2014 | Matsumoto, Nagano | - | - | H. Miyairi | LC519258 |
| 11 | HS. 001 | G | *Quercus acutissima* | -(2014) | Ina, Nagano | - | - | H. Sekine | LC519120 |
| 12 | TY. 026 | G | *Quercus acutissima* | 15-VII-2014 | Matsumoto, Nagano | - | - | T. Yamamoto | LC519121 |
| 13 | TY. 033 | G | *Quercus acutissima* | 15-VII-2014 | Matsumoto, Nagano | - | - | T. Yamamoto | LC519125 |
| 14 | TY. 034 | G | *Quercus acutissima* | 15-VII-2014 | Matsumoto, Nagano | - | - | T. Yamamoto | LC519126 |
| 15 | TY. 036 | G | *Quercus acutissima* | 15-VII-2014 | Matsumoto, Nagano | - | - | T. Yamamoto | LC519127 |
| 16 | TY. 039 | G | *Quercus acutissima* | 15-VII-2014 | Matsumoto, Nagano | - | - | T. Yamamoto | LC519129 |
| 17 | TY. 040 | G | *Quercus acutissima* | 15-VII-2014 | Matsumoto, Nagano | - | - | T. Yamamoto | LC519130 |
| 18 | TY. 042 | G | *Quercus acutissima* | 16-VII-2014 | Matsumoto, Nagano | - | - | T. Yamamoto | LC519132 |
| 19 | TY. 045 | G | *Quercus acutissima* | 16-VII-2014 | Matsumoto, Nagano | - | - | T. Yamamoto | LC519135 |
| 20 | TY. 048 | G | *Quercus acutissima* | 16-VII-2014 | Matsumoto, Nagano | - | - | T. Yamamoto | LC519137 |
| no. | DNA voucher # | Lineage | Host plant | Collection date | Location | Latitude | Longitude | Collectors | Genbank #COII |
| 22 | TY. 051 | G | *Quercus acutissima* | 20-VII-2014 | Azumino, Nagano | N36º20'05.31" | E137º51'26.90" | T. Yamamoto and M. Hattori | LC519139 |
| 23 | TY. 053 | G | *Quercus acutissima* | 20-VII-2014 | Azumino, Nagano | N36º20'05.31" | E137º51'26.90" | T. Yamamoto and M. Hattori | LC519140 |
| 24 | TY. 055 | G | *Quercus acutissima* | 20-VII-2014 | Azumino, Nagano | N36º19'43.06" | E137º49'44.64" | T. Yamamoto and M. Hattori | LC519150 |
| 25 | TY. 098 | G | *Quercus acutissima* | 7-IX-2014 | Higashichikuma, Nagano | N36º29'18.46" | E138º04'08.97" | T. Yamamoto and M. Hattori | LC519101 |
| 26 | TY. 099 | G | *Quercus acutissima* | 7-IX-2014 | Nagano, Nagano | N36º35'26.40" | E138º11'18.67" | T. Yamamoto and M. Hattori | LC519102 |
| 27 | TY. 100 | G | *Quercus acutissima* | 7-IX-2014 | Nagano, Nagano | N36º35'25.26" | E138º11'19.54" | T. Yamamoto and M. Hattori | LC519104 |
| 28 | TY. 101 | G | *Quercus acutissima* | 7-IX-2014 | Nagano, Nagano | N36º35'25.89" | E138º11'14.28" | T. Yamamoto and M. Hattori | LC519106 |
| 29 | TY. 105 | G | *Quercus acutissima* | 19-IX-2014 | Matsumoto, Nagano | N36º15'50.6" | E137º57'10.2" | T. Yamamoto | LC519107 |
| 30 | TY. 106 | G | *Quercus acutissima* | 19-IX-2014 | Matsumoto, Nagano | N36º15'50.6" | E137º57'10.2" | T. Yamamoto | LC519108 |
| 31 | TY. 107 | G | *Quercus acutissima* | 19-IX-2014 | Matsumoto, Nagano | N36º15'51.2" | E137º57'11.7" | T. Yamamoto | LC519110 |
| 32 | TY. 109 | G | *Quercus acutissima* | 19-IX-2014 | Matsumoto, Nagano | N36º15'24.6" | E137º57'53.3" | T. Yamamoto | LC519111 |
| 33 | TY. 110 | G | *Quercus acutissima* | 19-IX-2014 | Matsumoto, Nagano | N36º15'24.6" | E137º57'53.3" | T. Yamamoto | LC519174 |
| 34 | TY. 115 | G | *Quercus acutissima* | 24-IX-2014 | Takasaki, Gunma | N36º17'50.09" | E139º04'54.21" | T. Yamamoto | LC519176 |
| 35 | TY. 117 | G | *Quercus acutissima* | 25-IX-2014 | Takasaki, Gunma | N36º17'48.26" | E139º05'08.29" | T. Yamamoto | LC519177 |
| 36 | TY. 118 | G | *Quercus acutissima* | 25-IX-2014 | Higashimurayama, Tokyo | N35º46'07.07" | E139º27'03.20" | T. Yamamoto | LC519177 |
| 37 | TY. 163 | G | *Quercus acutissima* | 13-VII-2015 | Daimonjiyama, Kyoto | N35º01'26.04" | E135º48'10.34" | T. Yamamoto and M. Hattori | LC519213 |
| 38 | HY. 014 | G | *Quercus variabilis* | 13-X-2015 | Matsuyama, Ehime | - | - | H. Yoshitomi | - |
| 39 | TY. 065 | G | *Quercus variabilis* | 5-VIII-2014 | Nishio, Aichi | N34º52'42.41" | E137º05'03.65" | T. Yamamoto | LC519150 |
| 40 | TY. 146 | G | *Picea jezoensis*  var. *hondoensis* | 17-X-2014 | Matsumoto, Nagano | N36º15'34.85" | E137º59'29.04" | T. Yamamoto | LC519197 |
| 41 | TY. 096 | G | *Pinus densiflora* | 7-IX-2014 | Higashichikuma, Nagano | N36º29'15.02" | E138º04'13.20" | T. Yamamoto and M. Hattori | LC519100 |
| no. | DNA voucher # | Lineage | Host plant | Collection date | Location | Latitude | Longitude | Collectors | Genbank #COII |
| 43 | TY. 167 | G | *Pinus densiflora* | 26-IV-2016 | Daimonjiyama, Kyoto | N35º01'21.83" | E135º48'18.09" | T. Yamamoto | LC519213 |
|  |  |  |  |  |  |  |  |  |  |
| 45 | YM. 045 | G | *Pinus* sp. | 30-XI-2014 | Himeji, Hyogo | N34º52'09.4" | E134º32'38.4" | Y. Matsumoto | LC519246 |
| 46 | TK. 004 | H | *Quercus serrata* | 17-VI-2010 | Numazu, Shizuoka | - | - | T. Komatsu | LC519113 |
| 47 | TY. 151 | H | *Quercus serrata* | 6-VI-2015 | Sakura, Chiba | N35º43'22.83" | E140º13'15.89" | T. Yamamoto and M. Hattori | LC519201 |
| 48 | TY. 156 | H | *Quercus serrata* | 6-VI-2015 | Sakura, Chiba | N35º43'21.75" | E140º12'58.56" | T. Yamamoto and M. Hattori | LC519205 |
| 49 | TY. 162 | H | *Quercus serrata* | 13-VII-2015 | Daimonjiyama, Kyoto | N35º01'35.34" | E135º48'08.19" | T. Yamamoto and M. Hattori | - |
| 50 | YM. 026 | H | *Quercus serrata* | 12-X-2014 | Hachioji, Tokyo | N35º37'24.0" | E139º14'42.9" | Y. Matsumoto | LC519236 |
| 51 | HY. 010 | I (Outgroup) | *Chamaecyparis obtusa* | 15-V-2015 | Saijo, Ehime | - | - | H. Yoshitomi | - |
| 52 | HY. 002 | Outgroup | *Celtis sinensis* | 18-VIII-2012 | Matsuyama, Ehime | - | - | H. Yoshitomi | - |
| 53 | HY. 005 | Outgroup | *Ulmus davidiana*  var. *japonica* | 13-IX-2013 | Sapporo, Hokkaido | - | - | H. Yoshitomi | - |
| 54 | HY. 011 | Outgroup | *Fagus crenata* | 2-VIII-2015 | Matsuyama, Ehime | - | - | H. Yoshitomi | - |
