# Supplementary material for: Seasonal Migration in the Aphid Genus Stomaphis (Hemiptera: Aphididae): Discovery of Host Alternation Between Woody Plants in Subfamily Lachninae
Source: J Insect Sci. 2020 Sep 30;20(5):13. doi: 10.1093/jisesa/ieaa103 (PMC7583267; doi:10.1093/jisesa/ieaa103)
Supplement: ieaa103_suppl_Supplementary_Table_S2 [file ieaa103_suppl_supplementary_table_s2.docx]

Table S2. Aphid specimens used in the morphological analysis.

| No. | Voucher # | Number of measured individuals | DNA specimen voucher # | Host plant | Morph | Collection date | Location | Latitude | Longitude | Collector |
| --- | --- | --- | --- | --- | --- | --- | --- | --- | --- | --- |
| 1 | K15 | 2 | - | *Quercus acutissima* | Alate vivipara | 28-V-2016 | Matsumoto, Nagano | N36º15'35.2" | E137º59'28.7" | T. Yamamoto |
| 2 | K16 | 1 | TY. 048 | *Quercus acutissima* | Alate vivipara | 28-V-2016 | Matsumoto, Nagano | - | - | T. Yamamoto |
| 3 | K22 | 1 | TY. 163 | *Quercus acutissima* | Alate vivipara | 1-VI-2016 | Daimonjiyama, Kyoto | N35º01'26.04" | E135º48'10.34" | T. Yamamoto |
| 4 | K25 | 1 | TY. 048 | *Quercus acutissima* | Apterous vivipara | 10-VI-2016 | Matsumoto, Nagano | - | - | T. Yamamoto |
| 5 | K27 | 2 | TY. 118 | *Quercus acutissima* | Alate vivipara | 11-VII-2016 | Higashimurayama, Tokyo | N35º46'07.07" | E139º27'03.20" | T. Yamamoto |
| 6 | K32 | 5 | TY. 048 | *Quercus acutissima* | Alate vivipara | 5-X-2016 | Matsumoto, Nagano | - | - | T. Yamamoto |
| 7 | K35 | 3 | TY. 163 | *Quercus acutissima* | Apterous and Alate vivipara | 14-X-2016 | Daimonjiyama, Kyoto | N35º01'26.04" | E135º48'10.34" | T. Yamamoto |
| 8 | K36 | 1 | TY. 163 | *Quercus acutissima* | Alate vivipara | 14-X-2016 | Daimonjiyama, Kyoto | N35º01'26.04" | E135º48'10.34" | T. Yamamoto |
| 9 | K45 | 1 | - | *Quercus acutissima* | Alate vivipara | 9-VI-2017 | Matsumoto, Nagano | N36º15'35.2" | E137º59'28.7" | T. Yamamoto |
| 10 | K53 | 4 | TY. 163 | *Quercus acutissima* | Apterous vivipara | 3-IX-2018 | Daimonjiyama, Kyoto | N35º01'26.04" | E135º48'10.34" | T. Yamamoto |
| 11 | K56 | 5 | TY. 033 | *Quercus acutissima* | Apterous vivipara | 28-VIII-2018 | Matsumoto, Nagano | - | - | T. Yamamoto |
| 12 | K23 | 1 | TY. 167 | *Pinus densiflora* | Alate vivipara | 1-VI-2016 | Daimonjiyama, Kyoto | N35º01'21.83" | E135º48'18.09" | T. Yamamoto |
| No. | Voucher # | Number of measured individuals | DNA specimen voucher # | *Host plant* | Morph | Collection date | Location | Latitude | Longitude | Collector |
| 13 | K29 | 1 | YM. 001 | *Pinus densiflora* | Apterous vivipara | 10-VIII-2016 | Kisohukushima, Nagano | - | - | T. Yamamoto |
| 14 | K37 | 4 | TY. 167 | *Pinus densiflora* | Alate vivipara | 14-X-2016 | Daimonjiyama, Kyoto | N35º01'21.83" | E135º48'18.09" | T. Yamamoto |
| 16 | K40 | 5 | TY. 167 | *Pinus densiflora* | Alate vivipara and Ovipara | 7-XI-2016 | Daimonjiyama, Kyoto | N35º01'21.83" | E135º48'18.09" | T. Yamamoto |
| 17 | K42 | 1 | - | *Pinus densiflora* | Fundatrix | 26-V-2017 | Azumino, Nagano | N36º19'25.72" | E137º51'02.79" | T. Yamamoto |
| 18 | K43 | 2 | - | *Pinus densiflora* | Fundatrix | 26-V-2017 | Azumino, Nagano | N36º19'25.72" | E137º51'02.79" | T. Yamamoto |
| 19 | K44 | 3 | - | *Pinus densiflora* | Alate vivipara | 6-VI-2017 | Azumino, Nagano | N36º19'25.72" | E137º51'02.79" | T. Yamamoto |
| 20 | K20 | 1 | TY. 162 | *Quercus serrata* | Apterous vivipara | 1-VI-2016 | Daimonjiyama, Kyoto | N35º01'35.34" | E135º48'08.19" | T. Yamamoto |
| 21 | K21 | 1 | TY. 162 | *Quercus serrata* | Apterous vivipara | 1-VI-2016 | Daimonjiyama, Kyoto | N35º01'35.34" | E135º48'08.19" | T. Yamamoto |
| 22 | K3 | 2 | TY. 162 | *Quercus serrata* | Fundatrix | 26-IV-2016 | Daimonjiyama, Kyoto | N35º01'35.34" | E135º48'08.19" | T. Yamamoto |
| 23 | K34 | 3 | TY. 162 | *Quercus serrata* | Apterous vivipara | 14-X-2016 | Daimonjiyama, Kyoto | N35º01'35.34" | E135º48'08.19" | T. Yamamoto |
| 24 | K38 | 2 | TY. 162 | *Quercus serrata* | Apterous vivipara | 14-X-2016 | Daimonjiyama, Kyoto | N35º01'35.34" | E135º48'08.19" | T. Yamamoto |
| 25 | K39 | 3 | TY. 162 | *Quercus serrata* | Ovipara | 7-XI-2016 | Daimonjiyama, Kyoto | N35º01'35.34" | E135º48'08.19" | T. Yamamoto |
| 26 | K52 | 2 | TY. 162 | *Quercus serrata* | Apterous vivipara | 3-IX-2018 | Daimonjiyama, Kyoto | N35º01'35.34" | E135º48'08.19" | T. Yamamoto |
