# Supplementary material for: Seasonal Migration in the Aphid Genus Stomaphis (Hemiptera: Aphididae): Discovery of Host Alternation Between Woody Plants in Subfamily Lachninae
Source: J Insect Sci. 2020 Sep 30;20(5):13. doi: 10.1093/jisesa/ieaa103 (PMC7583267; doi:10.1093/jisesa/ieaa103)
Supplement: ieaa103_suppl_Supplementary_Table_S3 [file ieaa103_suppl_supplementary_table_s3.docx]

Table S3. Summary of the measurements of the morphological features of apterous viviparous females.

| Host plant | *Pinus densiflora* | *Quercus acutissima* | | | *Quercus serrata* | | |
| --- | --- | --- | --- | --- | --- | --- | --- |
| Morph | Apterous vivipara (*N* = 1) | Apterous vivipara (*N* = 12) | | | Apterous vivipara (*N* = 9) | | |
|  | one individual | Max | Min | Mean | Max | Min | Mean |
| Antennal segment I (mm) | 0.162 | 0.206 | 0.167 | 0.181 | 0.179 | 0.134 | 0.159 |
| Antennal segment II (mm) | 0.136 | 0.163 | 0.110 | 0.131 | 0.163 | 0.117 | 0.137 |
| Antennal segment III (mm) | 0.773 | 0.949 | 0.607 | 0.816 | 0.699 | 0.427 | 0.600 |
| Antennal segment IV (mm) | 0.295 | 0.344 | 0.241 | 0.311 | 0.270 | 0.176 | 0.235 |
| Antennal segment V (mm) | 0.328 | 0.387 | 0.286 | 0.333 | 0.335 | 0.225 | 0.293 |
| Antennal segment VI (mm) | 0.415 | 0.474 | 0.368 | 0.423 | 0.443 | 0.353 | 0.391 |
| Processus terminalis (mm) | 0.060 | 0.086 | 0.046 | 0.062 | 0.075 | 0.048 | 0.063 |
| Primary rhinarium (mm) | 0.053 | 0.068 | 0.037 | 0.049 | 0.060 | 0.040 | 0.049 |
| Middle tarsus I (mm) | 0.106 | 0.120 | 0.100 | 0.112 | 0.122 | 0.085 | 0.104 |
| Middle tarsus II (mm) | 0.255 | 0.290 | 0.237 | 0.264 | 0.234 | 0.187 | 0.214 |
| Hind tarsus I (mm) | 0.107 | 0.129 | 0.097 | 0.111 | 0.117 | 0.092 | 0.106 |
| Hind tarsus II (mm) | 0.322 | 0.366 | 0.289 | 0.331 | 0.302 | 0.240 | 0.272 |
| Rostral segment I (mm) | 4.805 | 5.575 | 4.688 | 5.247 | 5.792 | 4.337 | 5.155 |
| Rostral segment II (mm) | 5.165 | 5.942 | 4.458 | 5.346 | 5.554 | 3.925 | 4.985 |
| Rostral segment III (mm) | 0.726 | 0.818 | 0.694 | 0.765 | 0.734 | 0.583 | 0.647 |
| Rostral segment IV (mm) | 0.602 | 0.683 | 0.503 | 0.581 | 0.548 | 0.480 | 0.513 |
| Rostral segment V (mm) | 0.104 | 0.135 | 0.106 | 0.123 | 0.129 | 0.113 | 0.122 |
| Antenna I/II | 1.191 | 1.600 | 1.243 | 1.389 | 1.281 | 1.086 | 1.165 |
| Antenna III/II | 5.684 | 7.136 | 5.518 | 6.238 | 5.094 | 3.529 | 4.380 |
| Antenna III/IV | 2.620 | 2.967 | 2.415 | 2.628 | 2.893 | 2.243 | 2.564 |
| Antenna V/IV | 1.112 | 1.233 | 0.948 | 1.077 | 1.379 | 1.181 | 1.253 |
| Antenna VI/V | 1.265 | 1.415 | 1.164 | 1.272 | 1.622 | 1.054 | 1.352 |
| PT/Antenna VI | 0.145 | 0.189 | 0.116 | 0.146 | 0.205 | 0.127 | 0.161 |
| PT/PR | 1.132 | 1.625 | 0.838 | 1.300 | 1.667 | 0.917 | 1.278 |
| MT II/MT I | 2.406 | 2.573 | 2.175 | 2.373 | 2.326 | 1.872 | 2.056 |
| HT II/HT I | 3.009 | 3.309 | 2.773 | 2.981 | 2.849 | 2.265 | 2.568 |
| HT I/MT I | 1.009 | 1.111 | 0.897 | 1.000 | 1.232 | 0.893 | 1.023 |
| HT II/MT II | 1.263 | 1.310 | 1.219 | 1.254 | 1.368 | 1.175 | 1.273 |

Yellow shading indicates morphological parameter values that did not overlap between aphid individuals feeding on Q. acutissima or P. densiflora and those feeding on Q. serrata.

Abbreviations: MT - middle tarsus, HT - hind tarsus, PT - processus terminalis, PR - primary rhinarium
