# Supplementary material for: Seasonal Migration in the Aphid Genus Stomaphis (Hemiptera: Aphididae): Discovery of Host Alternation Between Woody Plants in Subfamily Lachninae
Source: J Insect Sci. 2020 Sep 30;20(5):13. doi: 10.1093/jisesa/ieaa103 (PMC7583267; doi:10.1093/jisesa/ieaa103)
Supplement: ieaa103_suppl_Supplementary_Table_S4 [file ieaa103_suppl_supplementary_table_s4.docx]

Table S4. Summary of measurements of the morphological characters of alate viviparous females.

| Host plant | *Pinus densiflora* | | | *Quercus acutissima* | | |
| --- | --- | --- | --- | --- | --- | --- |
| Morph | Alate vivipara (*N* = 10) | | | Alate vivipara (*N* = 13) | | |
|  | Max | Min | Mean | Max | Min | Mean |
| Antennal segment I (mm) | 0.176 | 0.138 | 0.163 | 0.208 | 0.144 | 0.172 |
| Antennal segment II (mm) | 0.153 | 0.124 | 0.136 | 0.151 | 0.120 | 0.140 |
| Antennal segment III (mm) | 0.860 | 0.650 | 0.765 | 0.912 | 0.667 | 0.788 |
| Antennal segment IV (mm) | 0.396 | 0.263 | 0.309 | 0.381 | 0.266 | 0.328 |
| Antennal segment V (mm) | 0.373 | 0.298 | 0.325 | 0.414 | 0.292 | 0.338 |
| Antennal segment VI (mm) | 0.458 | 0.373 | 0.418 | 0.492 | 0.359 | 0.431 |
| Processus terminalis (mm) | 0.072 | 0.046 | 0.061 | 0.084 | 0.040 | 0.059 |
| Primary rhinarium (mm) | 0.078 | 0.052 | 0.066 | 0.081 | 0.058 | 0.069 |
| Middle tarsus I (mm) | 0.117 | 0.090 | 0.104 | 0.130 | 0.089 | 0.109 |
| Middle tarsus II (mm) | 0.276 | 0.208 | 0.243 | 0.288 | 0.225 | 0.261 |
| Hind tarsus I (mm) | 0.118 | 0.085 | 0.105 | 0.133 | 0.095 | 0.111 |
| Hind tarsus II (mm) | 0.362 | 0.283 | 0.320 | 0.434 | 0.258 | 0.340 |
| Rostral segment I (mm) | 4.634 | 3.638 | 4.217 | 4.918 | 3.172 | 4.383 |
| Rostral segment II (mm) | 4.858 | 4.184 | 4.595 | 5.162 | 3.762 | 4.644 |
| Rostral segment III (mm) | 0.741 | 0.553 | 0.642 | 0.796 | 0.602 | 0.690 |
| Rostral segment IV (mm) | 0.574 | 0.496 | 0.528 | 0.582 | 0.406 | 0.538 |
| Rostral segment V (mm) | 0.136 | 0.105 | 0.122 | 0.147 | 0.106 | 0.118 |
| Antenna I/II | 1.293 | 1.113 | 1.201 | 1.612 | 1.061 | 1.229 |
| Antenna III/II | 6.266 | 5.242 | 5.639 | 6.362 | 4.935 | 5.620 |
| Antenna III/IV | 2.756 | 2.162 | 2.491 | 2.628 | 1.916 | 2.407 |
| Antenna V/IV | 1.235 | 0.912 | 1.061 | 1.192 | 0.890 | 1.034 |
| Antenna VI/V | 1.371 | 1.144 | 1.290 | 1.415 | 1.183 | 1.274 |
| PT/Antenna VI | 0.171 | 0.123 | 0.144 | 0.171 | 0.107 | 0.135 |
| PT/PR | 1.346 | 0.648 | 0.927 | 1.133 | 0.630 | 0.853 |
| MT II/MT I | 2.602 | 2.132 | 2.357 | 2.989 | 2.083 | 2.406 |
| HT II/HT I | 3.449 | 2.652 | 3.072 | 3.594 | 2.389 | 3.064 |
| HT I/MT I | 1.063 | 0.944 | 1.011 | 1.198 | 0.891 | 1.021 |
| HT II/MT II | 1.519 | 1.223 | 1.316 | 1.507 | 1.024 | 1.300 |

Yellow shading indicates morphological parameter values that did not overlap between aphid individuals feeding on Q. acutissima or P. densiflora and those feeding on Q. serrata.

Abbreviations: MT - middle tarsus, HT - hind tarsus, PT - processus terminalis, PR - primary rhinarium
