# Supplementary material for: Seasonal Migration in the Aphid Genus Stomaphis (Hemiptera: Aphididae): Discovery of Host Alternation Between Woody Plants in Subfamily Lachninae
Source: J Insect Sci. 2020 Sep 30;20(5):13. doi: 10.1093/jisesa/ieaa103 (PMC7583267; doi:10.1093/jisesa/ieaa103)
Supplement: ieaa103_suppl_Supplementary_Table_S5 [file ieaa103_suppl_supplementary_table_s5.docx]

Table S5. Summary of the measurements of the morphological features of fundatrices.

| Host plant | *Pinus densiflora* | | | *Quercus serrata* | | |
| --- | --- | --- | --- | --- | --- | --- |
| Morph | Fundatrix (*N* = 8) | | | Fundatrix (*N* = 2) | | |
|  | Max | Min | Mean | Max | Min | Mean |
| Antennal segment I (mm) | 0.165 | 0.144 | 0.158 | 0.157 | 0.144 | 0.151 |
| Antennal segment II (mm) | 0.151 | 0.108 | 0.134 | 0.142 | 0.136 | 0.139 |
| Antennal segment III (mm) | 0.743 | 0.552 | 0.682 | 0.474 | 0.470 | 0.472 |
| Antennal segment IV (mm) | 0.289 | 0.183 | 0.251 | 0.204 | 0.197 | 0.201 |
| Antennal segment V (mm) | 0.365 | 0.298 | 0.330 | 0.330 | 0.300 | 0.315 |
| Antennal segment VI (mm) | 0.380 | 0.311 | 0.352 | 0.343 | 0.339 | 0.341 |
| Processus terminalis (mm) | 0.061 | 0.036 | 0.049 | 0.067 | 0.063 | 0.065 |
| Primary rhinarium (mm) | 0.054 | 0.038 | 0.046 | 0.050 | 0.049 | 0.050 |
| Middle tarsus I (mm) | 0.105 | 0.094 | 0.098 | 0.105 | 0.098 | 0.102 |
| Middle tarsus II (mm) | 0.256 | 0.226 | 0.237 | 0.211 | 0.210 | 0.211 |
| Hind tarsus I (mm) | 0.109 | 0.089 | 0.101 | 0.113 | 0.109 | 0.111 |
| Hind tarsus II (mm) | 0.297 | 0.265 | 0.281 | 0.263 | 0.254 | 0.259 |
| Rostral segment I (mm) | 3.976 | 3.163 | 3.522 | 4.115 | 3.644 | 3.880 |
| Rostral segment II (mm) | 4.031 | 3.371 | 3.827 | 3.854 | 3.771 | 3.813 |
| Rostral segment III (mm) | 0.686 | 0.525 | 0.588 | 0.486 | 0.456 | 0.471 |
| Rostral segment IV (mm) | 0.537 | 0.453 | 0.496 | 0.487 | 0.479 | 0.483 |
| Rostral segment V (mm) | 0.132 | 0.091 | 0.112 | 0.111 | 0.100 | 0.106 |
| Antenna I/II | 1.528 | 1.053 | 1.193 | 1.154 | 1.014 | 1.084 |
| Antenna III/II | 5.488 | 4.759 | 5.094 | 3.456 | 3.338 | 3.397 |
| Antenna III/IV | 3.016 | 2.502 | 2.734 | 2.386 | 2.324 | 2.355 |
| Antenna V/IV | 1.628 | 1.188 | 1.330 | 1.675 | 1.471 | 1.573 |
| Antenna VI/V | 1.235 | 0.975 | 1.071 | 1.143 | 1.027 | 1.085 |
| PT/Antenna VI | 0.166 | 0.102 | 0.138 | 0.195 | 0.186 | 0.191 |
| PT/PR | 1.326 | 0.667 | 1.067 | 1.340 | 1.286 | 1.313 |
| MT II/MT I | 2.500 | 2.353 | 2.415 | 2.153 | 2.000 | 2.077 |
| HT II/HT I | 3.337 | 2.477 | 2.805 | 2.413 | 2.248 | 2.330 |
| HT I/MT I | 1.126 | 0.848 | 1.026 | 1.112 | 1.076 | 1.094 |
| HT II/MT II | 1.233 | 1.158 | 1.183 | 1.246 | 1.210 | 1.228 |

Yellow shading indicates morphological parameter values that did not overlap between aphid individuals feeding on Q. acutissima or P. densiflora and those feeding on Q. serrata.

Abbreviations: MT - middle tarsus, HT - hind tarsus, PT - processus terminalis, PR - primary rhinarium
