# Supplementary material for: Seasonal Migration in the Aphid Genus Stomaphis (Hemiptera: Aphididae): Discovery of Host Alternation Between Woody Plants in Subfamily Lachninae
Source: J Insect Sci. 2020 Sep 30;20(5):13. doi: 10.1093/jisesa/ieaa103 (PMC7583267; doi:10.1093/jisesa/ieaa103)
Supplement: ieaa103_suppl_Supplementary_Table_S6 [file ieaa103_suppl_supplementary_table_s6.docx]

Table S6. Summary of the measurements of the morphological features of oviparae.

| Host plant | *Pinus densiflora* | | | *Quercus serrata* | | |
| --- | --- | --- | --- | --- | --- | --- |
| Morph | Ovipara (*N* = 3) | | | Ovipara (*N* = 3) | | |
|  | Max | Min | Mean | Max | Min | Mean |
| Antennal segment I (mm) | 0.161 | 0.149 | 0.153 | 0.163 | 0.147 | 0.157 |
| Antennal segment II (mm) | 0.137 | 0.123 | 0.129 | 0.150 | 0.108 | 0.130 |
| Antennal segment III (mm) | 0.772 | 0.737 | 0.760 | 0.659 | 0.518 | 0.590 |
| Antennal segment IV (mm) | 0.301 | 0.280 | 0.289 | 0.252 | 0.214 | 0.232 |
| Antennal segment V (mm) | 0.332 | 0.306 | 0.316 | 0.310 | 0.265 | 0.283 |
| Antennal segment VI (mm) | 0.437 | 0.411 | 0.424 | 0.422 | 0.378 | 0.405 |
| Processus terminalis (mm) | 0.080 | 0.066 | 0.074 | 0.069 | 0.058 | 0.064 |
| Primary rhinarium (mm) | 0.049 | 0.043 | 0.046 | 0.063 | 0.050 | 0.058 |
| Middle tarsus I (mm) | 0.100 | 0.084 | 0.094 | 0.123 | 0.098 | 0.111 |
| Middle tarsus II (mm) | 0.243 | 0.238 | 0.240 | 0.226 | 0.202 | 0.216 |
| Hind tarsus I (mm) | 0.108 | 0.094 | 0.100 | 0.128 | 0.100 | 0.117 |
| Hind tarsus II (mm) | 0.311 | 0.290 | 0.301 | 0.299 | 0.269 | 0.288 |
| Rostral segment I (mm) | 3.964 | 3.796 | 3.894 | 5.142 | 4.673 | 4.868 |
| Rostral segment II (mm) | 4.244 | 3.989 | 4.138 | 5.128 | 4.375 | 4.736 |
| Rostral segment III (mm) | 0.640 | 0.613 | 0.630 | 0.688 | 0.631 | 0.660 |
| Rostral segment IV (mm) | 0.494 | 0.462 | 0.480 | 0.529 | 0.529 | 0.529 |
| Rostral segment V (mm) | 0.123 | 0.108 | 0.114 | 0.134 | 0.127 | 0.131 |
| Antenna I/II | 1.211 | 1.173 | 1.187 | 1.361 | 1.067 | 1.218 |
| Antenna III/II | 6.071 | 5.635 | 5.899 | 4.796 | 4.393 | 4.549 |
| Antenna III/IV | 2.699 | 2.561 | 2.631 | 2.615 | 2.421 | 2.538 |
| Antenna V/IV | 1.161 | 1.017 | 1.094 | 1.238 | 1.191 | 1.220 |
| Antenna VI/V | 1.375 | 1.316 | 1.345 | 1.511 | 1.361 | 1.433 |
| PT/Antenna VI | 0.183 | 0.161 | 0.174 | 0.164 | 0.153 | 0.158 |
| PT/PR | 1.767 | 1.435 | 1.612 | 1.300 | 0.921 | 1.117 |
| MT II/MT I | 2.893 | 2.380 | 2.567 | 2.061 | 1.837 | 1.951 |
| HT II/HT I | 3.309 | 2.787 | 3.018 | 2.690 | 2.336 | 2.477 |
| HT I/MT I | 1.119 | 1.000 | 1.066 | 1.088 | 1.020 | 1.050 |
| HT II/MT II | 1.280 | 1.218 | 1.254 | 1.339 | 1.323 | 1.331 |

Yellow shading indicates morphological parameter values that did not overlap between aphid individuals feeding on Q. acutissima or P. densiflora and those feeding on Q. serrata.

Abbreviations: MT - middle tarsus, HT - hind tarsus, PT - processus terminalis, PR - primary rhinarium
