# Supplementary figures and images for: Seasonal Migration in the Aphid Genus Stomaphis (Hemiptera: Aphididae): Discovery of Host Alternation Between Woody Plants in Subfamily Lachninae
Source: J Insect Sci. 2020 Sep 30;20(5):13. doi: 10.1093/jisesa/ieaa103 (PMC7583267; doi:10.1093/jisesa/ieaa103)

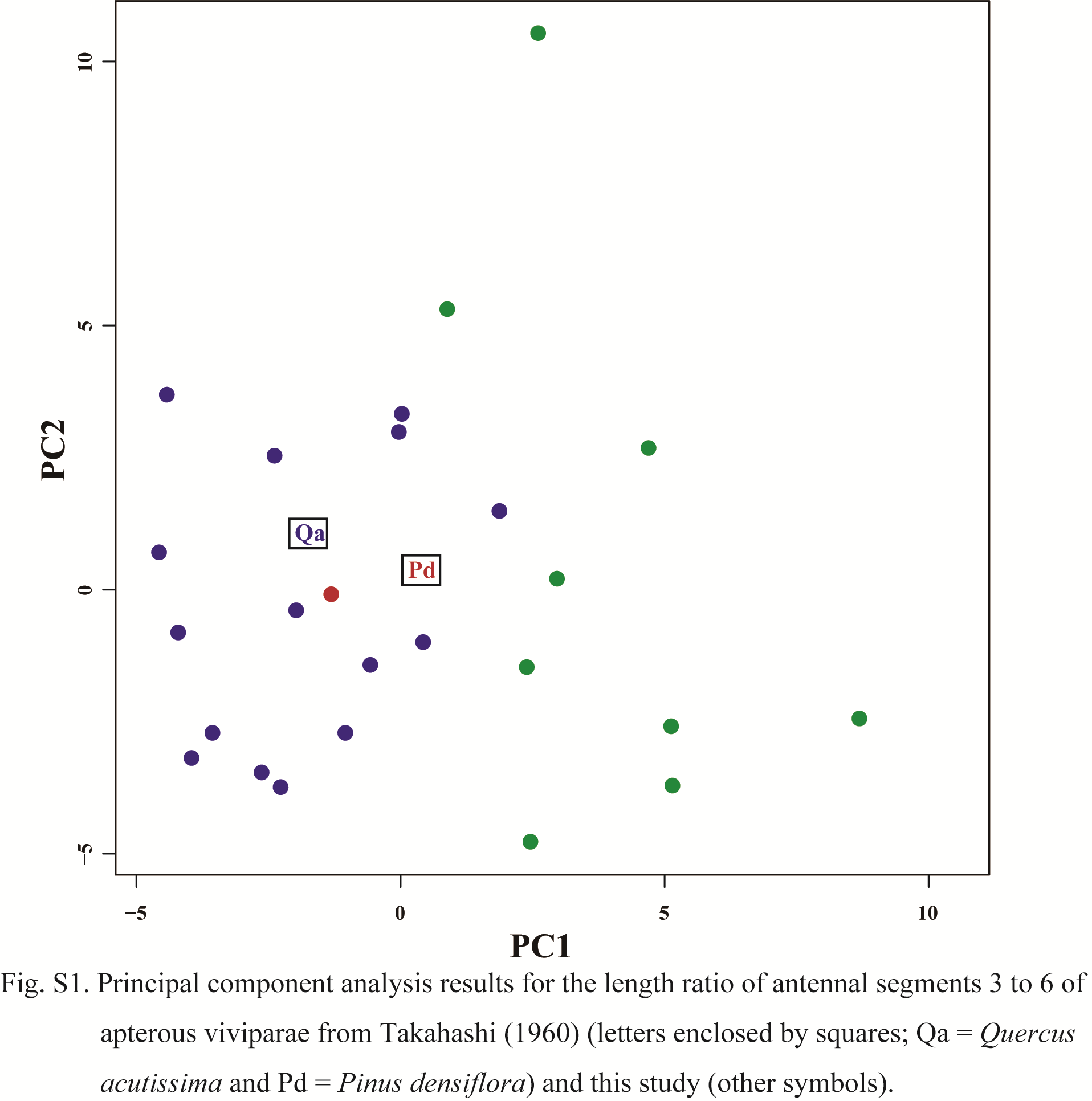

Supplement: ieaa103_suppl_Supplementary_Figure_S1 [file ieaa103_suppl_supplementary_figure_s1.png]
